# *ATM* gene mutations in sporadic breast cancer patients from Brazil

**DOI:** 10.1186/s40064-015-0787-z

**Published:** 2015-01-15

**Authors:** Flavia Rotea Mangone, Elisabete C Miracca, Harriet E Feilotter, Lois M Mulligan, Maria Aparecida Nagai

**Affiliations:** Laboratory of Molecular Genetics, Center for Translational Research in Oncology, Av Dr Arnaldo, 251, 8th Floor, CEP 01246-000 São Paulo, Brazil; Department of Pathology and Molecular Medicine, Richardson Laboratory, Queen’s University, 88 Stuart Street, Kingston, Ontario K7L 3N6 Canada; Department of Pathology and Molecular Medicine, Cancer Research Institute, Queen’s University, Botterell Hall, 10 Stuart Street, Kingston, Ontario K7L 3N6 Canada; Discipline of Oncology, Department of Radiology and Oncology, Faculty of Medicine, University of São Paulo, Av Dr Arnaldo, 455, 4th Floor, CEP 01246-903 São Paulo, Brazil

**Keywords:** Breast cancer, *ATM* gene, Mutations, Polymorphisms

## Abstract

**Purpose:**

The Ataxia-telangiectasia mutated (*ATM*) gene encodes a multifunctional kinase, which is linked to important cellular functions. Women heterozygous for *ATM* mutations have an estimated relative risk of developing breast cancer of 3.8. However, the pattern of *ATM* mutations and their role in breast cancer etiology has been controversial and remains unclear. In the present study, we investigated the frequency and spectrum of *ATM* mutations in a series of sporadic breast cancers and controls from the Brazilian population.

**Methods:**

Using PCR-Single Strand Conformation Polymorphism (SSCP) analysis and direct DNA sequencing, we screened a panel of 100 consecutive, unselected sporadic breast tumors and 100 matched controls for all 62 coding exons and flanking introns of the *ATM* gene.

**Results:**

Several polymorphisms were detected in 12 of the 62 coding exons of the *ATM* gene. These polymorphisms were observed in both breast cancer patients and the control population. In addition, evidence of potential *ATM* mutations was observed in 7 of the 100 breast cancer cases analyzed. These potential mutations included six missense variants found in exon 13 (p.L546V), exon 14 (p.P604S), exon 20 (p.T935R), exon 42 (p.G2023R), exon 49 (p.L2307F), and exon 50 (p.L2332P) and one nonsense mutation in exon 39 (p.R1882X), which was predicted to generate a truncated protein.

**Conclusions:**

Our results corroborate the hypothesis that sporadic breast tumors may occur in carriers of low penetrance *ATM* mutant alleles and these mutations confer different levels of breast cancer risk.

**Electronic supplementary material:**

The online version of this article (doi:10.1186/s40064-015-0787-z) contains supplementary material, which is available to authorized users.

## Background

The Ataxia-telangiectasia mutated (*ATM*) gene encodes a multifunctional kinase, which is linked to important cellular functions (Derheimer and Kastan 2010; Shiloh and Ziv 2013). Individuals carrying two *ATM* alleles with loss-of-function mutations are affected with Ataxia-Telangiectasia (A-T), which is an autosomal recessive syndrome characterized by progressive cerebellar ataxia, oculocutaneous telangiectasia, immunodeficiency, hypersensitivity to ionizing radiation, and cancer susceptibility (Gatti et al. 1991; Savitsky et al. 1995a; Ambrose and Gatti 2013). The estimated incidence of A-T is 1:40,000 to 1:100,000 live births (Easton, 1994). Patients with A-T have an increased risk of developing cancer compared with non-A-T individuals (Taylor and Byrd 2005; Mavrou et al. 2008). The frequency of individuals heterozygous for a mutation in *ATM* is estimated to be 0.5 - 1% in the general population (Swift et al. 1986; Easton 1994). Individuals heterozygous for A-T mutations, who are phenotypically normal, may exhibit increased radio- and chemo-sensitivity and an increased relative risk of carcinogenesis (Swift et al. 1991; West et al. 1995). An increased relative risk of breast cancer was identified for female A-T heterozygotes; while, the proportion of sporadic breast cancers in the general population that is due to *ATM* mutations remains controversial (Swift et al. 1987; Ahmed and Rahman 2006; Mavrou et al. 2008). Somatically occurring *ATM* mutations are also prevalent in a number of sporadic human cancers, most notably leukemias and carcinomas of the breast and lung (Cremona and Behrens, 2014).

The *ATM* gene is located on chromosome 11q22-q23, spanning approximately 150 kb of genomic DNA (Savitsky et al. 1995a; Savitsky et al. 1995b). *ATM* is structurally complex, containing 66 exons, and encodes a protein of 3056 amino acids (~350 kDa) that is related to the phosphatidylinositol 3-kinase (PI3-kinase) superfamily (Savitsky et al. 1995a, Lavin et al. 1995). The COOH-terminal region of the ATM protein shows similarity to several checkpoint/damage response proteins from other organisms, including *TOR1* and *TOR2*, *TEL1p*, *ATR*, *RAD3p*, *MEC1pC*, *MEI-41*, and *DNA-PK* (Mavrou et al. 2008; Bensimon et al. 2011). *ATM*-knockout mice show A-T-like phenotypes, including neurological abnormalities, immunodeficiency, chromosomal instability, radiosensitivity, and the development of lymphomas (Xu et al. 1996; Barlow et al. 1996; Liao and Van Dyke. 1999; Deng and Brodie 2001; Shiloh and Ziv 2012). *ATM* belongs to the class of caretaker tumor suppressor genes that defend genome integrity (Levitt and Hickson 2002). Irradiated A-T cells had defects in the activation of the G1/S, S phase, and G2 checkpoints (Beamish and Lavin 1994; Derheimer and Kastan 2010). ATM is believed to be involved in multiple cellular processes that occur in response to DNA damage, including cell cycle check point control, DNA repair, and apoptosis (Derheimer and Kastan 2010; Shiloh and Ziv 2013). ATM is one of the main sensors for DNA damage evoked by genotoxic agents, such as ionizing radiation (Rotman and Shiloh 1999; Thompson and Schild 2002; Liang et al. 2009). Members of the PI3-kinase superfamily of serine/threonine protein kinases, such as ATM and DNA-dependent protein kinase (DNA-PK) have been implicated in cell cycle checkpoint control in response to DNA damage (Derheimer and Kastan 2010; Sperka et al. 2012). ATM is considered an important transducer of DNA damage signaling via phosphorylation of downstream effectors, which results in a kinase cascade that works to promote genetic stability (Canman and Lim 1998; Khanna et al. 1998; Khosravi et al. 1999; Cortez et al. 1999; Li et al. 2000; Maya et al. 2001).

The size and complexity of the *ATM* gene make the search for mutations and identification of genotype-phenotype associations difficult, especially for the missense mutations primarily identified in heterozygous subjects. The nature and distribution of the *ATM* mutations found in A-T patients are variable with no specific hot spot (Yuille and Coignet 1998; Sandoval et al. 1999; Mavrou et al. 2008). More than 80% of A-T patients have ATM-truncating mutations; however, exon-skipping and missense mutations are also observed (Renwick et al. 2006; Mavrou et al. 2008). Detected mutations are believed to cause structural or functional effects on the final protein, abolishing ATM function. On the other hand, the majority of *ATM* variants described so far in sporadic leukemia and breast cancers are missense base substitutions (Prokopcova et al. 2007; Broeks et al. 2008), making it difficult to distinguish missense mutations that affect protein function from sequence polymorphisms with no effect on normal ATM protein activity.

While the spectrum of *ATM* mutations in classical A-T patients is well established, the pattern of *ATM* mutations and their role in breast cancer etiology remains unclear (Broeks et al. 2008; Milne 2009). The aim of the present study was to investigate the frequency and the spectrum of *ATM* mutations in a series of sporadic breast cancer and control samples from the Brazilian population.

## Results

DNA samples from a series of 100 breast cancer patients (tumor and normal tissues) and 100 control individuals were screened for *ATM* gene mutations. Sixty-four sets of primers were used to amplify all coding exons and flanking intron sequences of the *ATM* gene (Vorechovsky et al. 1996). The amplified DNA fragments were screened by PCR-Single Strand Conformation Polymorphism (SSCP) analysis and the samples showing band mobility shifts were further analyzed by direct DNA sequencing. This analysis led to the identification of 24 distinct sequence variants (Tables 1 and 2). The polymorphism p.P1054R (Table 1), which has been implicated in breast cancer and linked to F858L (Larson et al. 1997–1998; Fletcher et al., 2010), could not be detected by SSCP screening. The presence of this polymorphism in our population could be confirmed only by re-sequencing some of the breast cancer cases and controls showing the polymorphism p.F858L (Additional file 1); therefore, the SSCP technique had low sensitivity for this mutation. The majority of variants observed were base substitutions within the *ATM* coding region. One base substitution, one deletion, and two insertions were observed in the introns of the *ATM* gene.

**Table 1:** **ATM gene variants detected in controls and breast cancer patients**

**Position Exon/Intron**	**Nucleotide change**	**Residue**	**Variation type**	Functional impact*
7	c.378 T > A	p.D126E	Non-Synonymous	Neutral
7	c.1287-18delT	N/A	Non-coding	-
9	c.735C > T	p.V245V	Synonymous	Neutral
13	c.1744 T > C	p.F582L	Non-Synonymous	Neutral
15	c.2119 T > C	p.S707P	Non-Synonymous	Neutral
16	c.2193C > T	p.Y731Y	Synonymous	Neutral
18	c.2442C > A	p.D814E	Non-Synonymous	Neutral
19	c.2572 T > C	p.F858L	Non-Synonymous	Damaging
20	c.2685A > G	p.L895L	Synonymous	Neutral
20	c.2805G > C	p.T935T	Synonymous	Neutral
23	c.3118A > G	p.M1040V	Non-Synonymous	Neutral
24	c.3161C > G	p.P1054R	Non-Synonymous	Damaging
32	c.4578C > T	p.P1526P	Synonymous	Neutral
39	c.5557G > A	p.D1853N	Non-Synonymous	Neutral
55	c.8536 + 13insT	N/A	Non-coding	-
56	c.8653 + 30insT	N/A	Non-coding	-
62	c.9372 + 8A > C	N/A	Non-coding	-

http://genetics.bwh.harvard.edu/pph2/; http://provean.jcvi.org/genome_submit_2.php;

http://chromium.liacs.nl/LOVD2/home.php?select_db=ATM (Last update January 2012); http://www.ncbi.nlm.nih.gov/projects/SNP/snp_ref.cgi?geneId=472; http://www.ensembl.org/Homo_sapiens/Gene/Variation_Gene/Table?db=core;g=ENSG00000149311;r=11:108093211–108239829; * http://www.1000genomes.org/1000-genomes-browsers; http://evs.gs.washington.edu/EVS/.

**Table 2:** **Potential mutations in the** ***ATM*** **gene identified in Brazilian breast cancer patients**

**Exon position**	**Nucleotide change**	**Residue**	Functional impact*	**LOH**	**Sample**	Age*** (years)	**Clinical stage**
13	c.1636C > G	p.L546V	Damaging	No	M227	46	I
14	c.1810C > T	p.P604S	Damaging	Yes**	M231	50	IV
20	c.2804C > G	p.T935R	Neutral	Yes**	M214	51	III
39	c.5647C > T	p.R1882X	Protein truncation	Yes**	M115	75	III
42	c.6067G > A	p.G2023R	Damaging	No	M294	45	II
49	c.6919C > T	p.L2307F	Damaging	No	72TL	67	II
				Yes	M214	51	
50	c.6995 T > C	p.L2332P	Neutral	Yes	112TL	25	II
				No	M227	46	

http://chromium.liacs.nl/LOVD2/home.php?select_db=ATM (Last update January 2012); http://www.ncbi.nlm.nih.gov/projects/SNP/snp_ref.cgi?geneId=472;

http://www.ensembl.org/Homo_sapiens/Gene/Variation_Gene/Table?db=core;g=ENSG00000149311;r=11:108093211–108239829; http://www.1000genomes.org/1000-genomes-browsers.

*http://genetics.bwh.harvard.edu/pph2/; http://provean.jcvi.org/genome_submit_2.php.

**LOH, indicates loss of the wild type allele.

***age at diagnosis.

Seventeen of the *ATM* variants were detected in DNA samples from both breast cancer patients and the control population and were, thus, classified as polymorphisms. The polymorphisms observed in patients and controls are summarized in Table 1. Among these, five of the base substitutions were synonymous changes that did not affect amino acid sequences and 8 were non-synonymous changes (Table 1).

Seven unique DNA sequence variants were observed exclusively in samples from breast cancer patients and were classified as potential *ATM* mutations (Table 2). The alterations observed were distributed throughout the *ATM* coding sequence with no specific clustering, which would have suggested no mutational hot spot. The spectrum of the *ATM* variants observed only in breast tumors consisted of six missense mutations affecting exons 13, 14, 20, 42, 49, and 50 and one nonsense mutation affecting exon 39. Three of the base substitutions resulted in conservative amino acid changes (p.L546V, case M227; p.P604S, case M231; and p.L2307F, cases 72TL and M214) and three led to non-conservative changes (p.T935R, case M214; p.G2023R, case M294; and p.L2332P, cases 112TL and M214). Analyses of DNA from Case M115 identified a nonsense mutation in exon 39 (p.R1882X), which was not previously reported in sporadic breast cancer patients and was predicted to lead to protein truncation (Table 2). Two breast tumors demonstrated two distinct *ATM* variants that were classified as potential causal missense mutations, but several breast tumors and controls displayed multiple *ATM* variants classified as polymorphisms. The SIFT and PolyPhen algorithms were used to evaluate the potential functional effect of the missense variants, which could be classified as damaging (potential disease-causing mutation) or neutral variants (Tables 1 and 2). Two of the 13 (15.4%) variants identified in both control and breast cancer patients and 4 of the 6 (67%) potential missense mutations identified only in breast cancer patients were classified as damaging.

**Figure 1:**
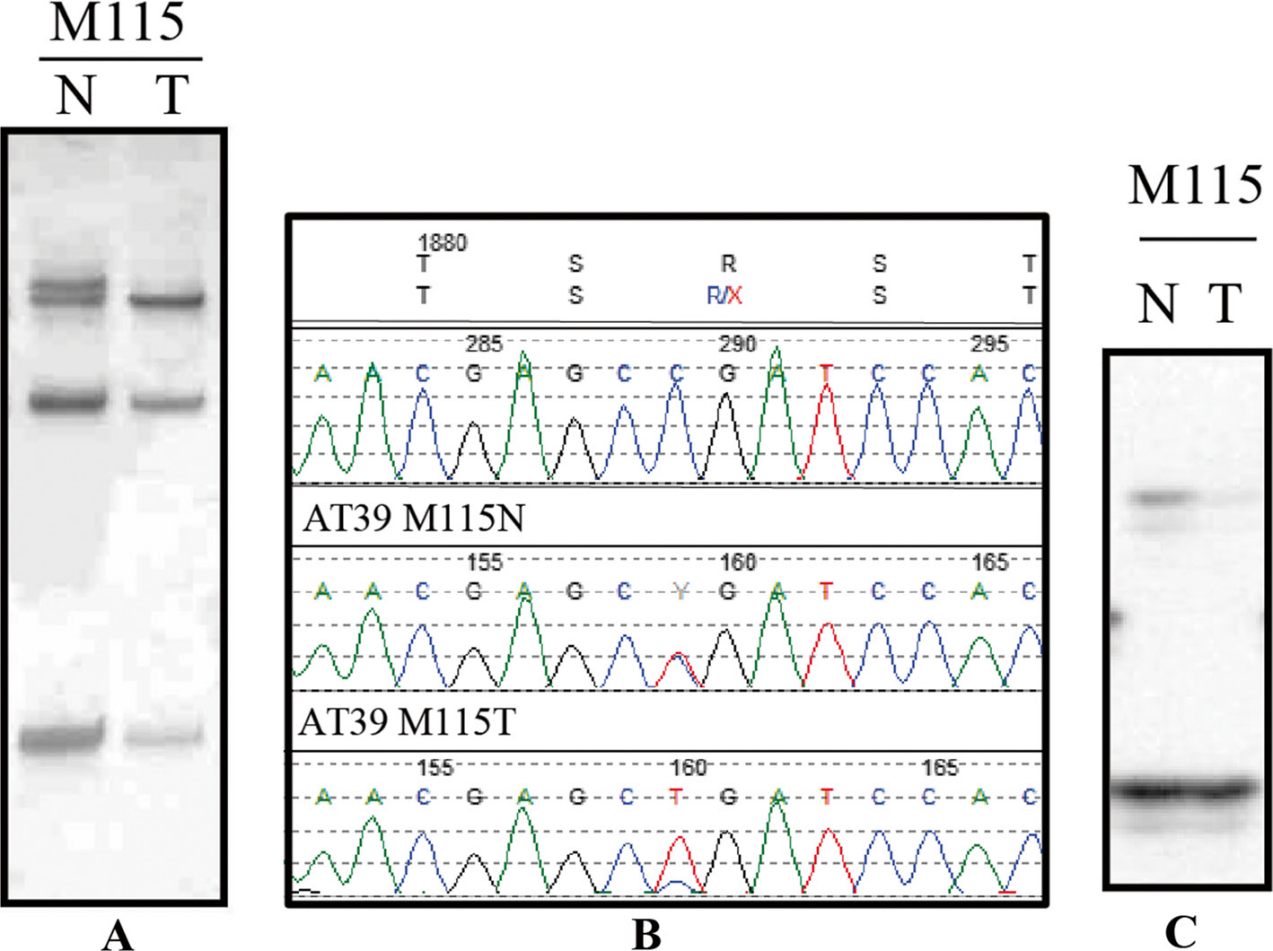
**ATM nonsense mutation in a sporadic breast cancer patient. A**, SSCP analysis of exon 39 (case M115); **B**, sequence profile for exon 39 showing a nonsense mutation R1882X; **C**, loss of heterozygosity at marker D11S1818; N, normal DNA; T, tumor DNA.

**Figure 2:**
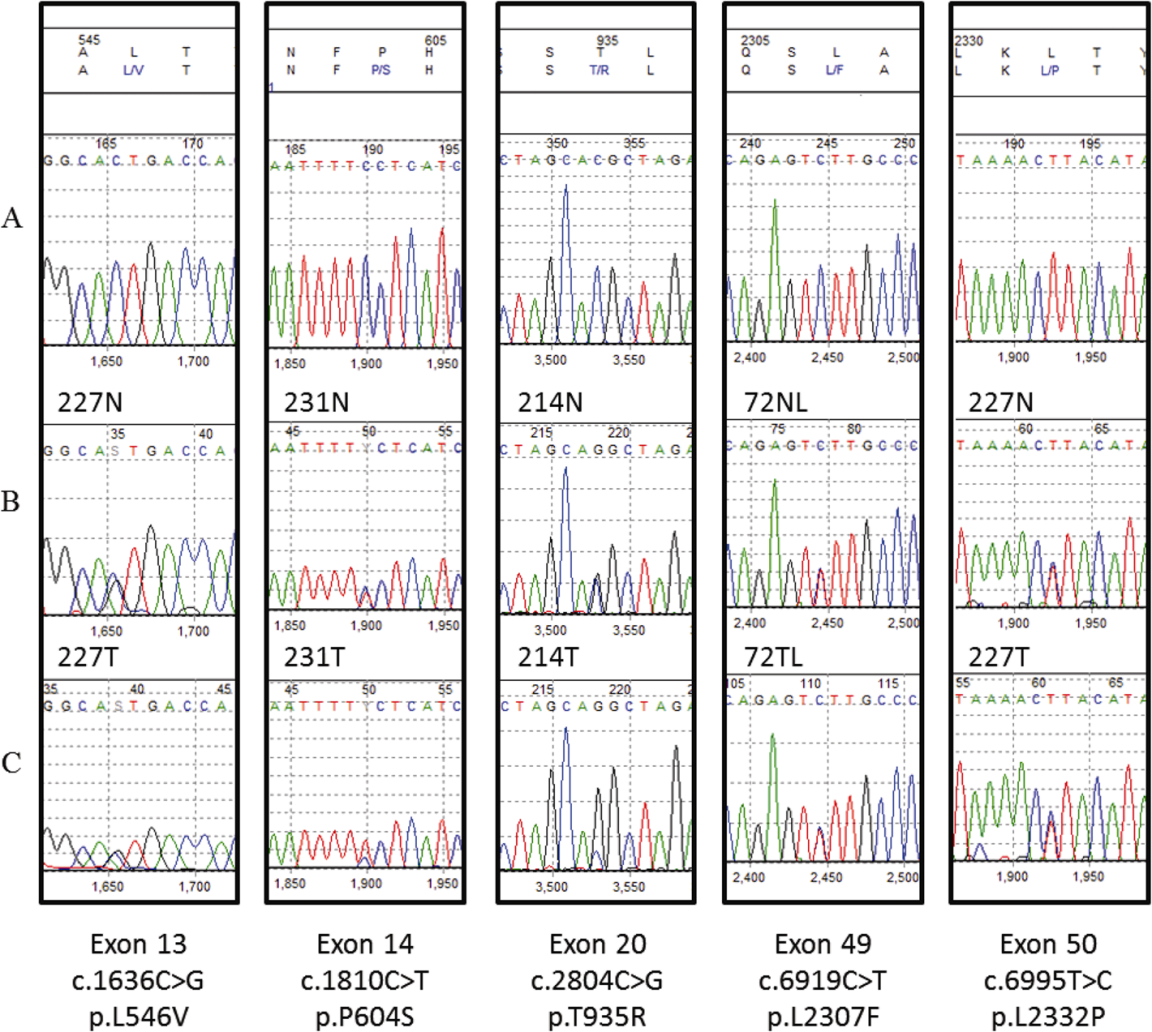
**Representative examples of the sequencing analysis of potential ATM mutations in sporadic breast cancer patients. A**, upper panel: reference sequence; **B**, middle panel: sequencing electropherograms from normal tissue samples from breast cancer patients (227 N, 231 N, 214 N, and 72NL); **C**, lower panel: sequencing electropherograms from tumor tissue samples from breast cancer patients (227 T, 231 T, 214 T, and 72TL). Case 227 showed two potential ATM missense mutations without evidence of LOH; Cases 214 and 231 showed potential ATM mutations with evidence of loss of the wild type allele.

DNA samples from the primary breast cancer patients were also examined for the occurrence of loss of heterozygosity (LOH) for markers D11S897, D11S1818, and D11S2000 that span approximately 10 cM of the chromosomal region 11q22-23 where the *ATM* gene is located. Allelic loss was detected in 36/90 (40%) tumors that were informative (heterozygous) for at least one of the markers examined. The frequency of allelic loss at each of the markers analyzed is listed in Table 3. Case M115 had the nonsense mutation in exon 39 (p.R1882X) as well as LOH for marker D11S1818 (Figure 1), suggesting that ATM function was abolished in this tumor. Three of the tumors with potential *ATM* mutations also showed LOH for the microsatellite markers on chromosomal region 11q23 (Table 2). However, the microsatellite markers used were not located within the *ATM* gene. We sequenced all DNA samples from normal and tumor tissues that had potential *ATM* mutations (Table 2). Sequencing results showed evidence of wild type allele loss in cases M214 and M231 harboring potential mutations (Figure 2) and in case M115 with a nonsense mutation. In addition, two patients showed two missense variants, classified as potential *ATM* mutations. Each patient harbor one variant classified as deleterious based on the prediction of functional impact (M214, p.L2307F; M227, p.L546V) and one variant predicted to be neutral (M214, p.T935R; M227, p.L2332P). We also identified three tumors harboring *ATM* variants classified as polymorphisms, such as p.S707P, p.F858L, and p.M1040V, with evidence of wild type allele loss.

**Table 3:** **Summary of Loss of Heterozygosity (LOH) on chromosome 11q in primary breast tumors**

**Locus**	**No. of cases analyzed**	Allelic loss/informative cases (%)*
D11S897	100	17/71 (24)
D11S1818	100	26/69 (38)
D11S2000	100	22/84 (26)

*Informative cases: individuals heterozygous for the microsatellite marker examined.

## Discussion

Epidemiological studies have suggested that A-T heterozygous individuals are at increased relative risk for cancer, especially breast cancer (Thompson et al. 2005; Ahmed and Rahman 2006). Hence, several studies have investigated the frequency, relative risk, and clinical significance of *ATM* mutations in breast cancer patients (Ahmed and Rahman 2006; Tavtigian et al. 2009; Graña et al. 2011). In the present study, we examined DNA from a series of 100 breast cancer patients without a family history of breast cancer and 100 healthy individuals for *ATM* gene mutations. To the best of our knowledge, this is the first study to investigate the occurrence of *ATM* mutations in a Brazilian breast cancer population. We were able to identify variants occurring only in the breast cancer population. Overall, 7% of the patient population had *ATM* gene alterations classified as potential mutations, with the majority being missense variants. Low penetrance, potentially deleterious *ATM* missense variants, which were associated with an increased risk for breast cancer, have been found in other breast cancer populations (Scott et al. 2002; Thorstenson et al. 2003; Graña et al. 2011). Using SIFT and PolyPhen tools to predict the possible impact of the amino acid changes on the ATM function, we found 4 of the 6 variants identified only in breast cancer patients could be classified as potentially deleterious mutations that might impair ATM function, suggesting a causality to the disease. Further experimental and clinical studies are needed to better understand the association of these variants with breast cancer risk. In addition, we also identified a sporadic breast cancer patient carrying a nonsense mutation, with loss of the wild-type allele, which could be a biologically significant *ATM* mutation. Although this variant, p.R1882X, has been described in Brazilian A-T patients (Mitui et al., 2003; Coutinho et al. 2004); to date, it has not been described in sporadic breast cancer patients.

To our knowledge the exact prevalence of A-T heterozygotes in the Brazilian population is unknown. However, there was a high prevalence for nonsense, splicing site, or frameshift *ATM* mutations in 27 Brazilian families with classical A-T, and these led to truncated proteins (Coutinho et al. 2004). We identified one individual carrying a nonsense mutation that was predicted to generate a truncated protein, a phenomenon frequently observed in classical A-T (Sandoval et al. 1999; Demuth et al. 2011). This finding is consistent with the frequency of 0.2 – 1% of A-T carriers in the general population, as predicted from epidemiological studies (Easton, 1994). The breast cancer patient carrying a nonsense mutation was constitutively heterozygous for the mutation, but a DNA sample from her tumor showed LOH for the wild-type allele, which is consistent with the tumor suppressor role predicted for the *ATM* gene in the pathogenesis of breast cancer. We identified two patients with two missense variants classified as potential *ATM* mutations*.* Each patient harbored one variant classified as deleterious (M214, pL2307F; M227, pL546V) and another variant classified as neutral (M214, p.T935R; M227, pL2332P). In addition, we identified some tumors with *ATM* variants classified as polymorphisms with evidence of LOH of the wild type allele, suggesting there was selection of the polymorphic allele during the tumorigenic process. Loss of the wild-type allele of the *ATM* gene has also been reported in tumors of individuals with familial breast cancer (Bay et al. 1999) and early-onset breast cancer (Izatt et al. 1999). Reduced levels of *ATM* mRNA and protein have also been reported in sporadic breast tumors, implicating *ATM* gene inactivation in the development and progression of breast cancer (Ding et al. 2004; Bhattacharya et al. 2013; Bueno et al. 2014). There has been a high frequency of DNA methylation observed in advanced breast tumors and attributed to reduced ATM expression and function (Vo et al. 2004). Furthermore, recent studies demonstrated that over-expression of microRNAs, such as miR181 and miR421, may be involved in ATM down-regulation in breast cancer (Fang et al. 2010; Bueno et al. 2014).

More than 70% of the *ATM* mutations described in classical A-T families are frameshift or nonsense mutations leading to protein truncation (Cavaciutti et al. 2005; Goldgar et al. 2011). To date, most of the *ATM* mutations observed in breast cancer patients, including the data reported here, are missense mutations distributed throughout the *ATM* gene, but mainly outside of the PI-3 K domain or other putative functional domains defined by sequence similarity (Savitsky et al. 1995a; Bosotti et al. 2000; Thorstenson et al. 2001; Tavtigian et al. 2009). Thus, it is difficult to define clear genotype-phenotype associations, and the clinical implications of these mutations remain unknown.

*In vitro* studies have shown that lymphocytes from *ATM* mutation-carriers are more radiosensitive than lymphocytes from normal donors (West et al. 1995), suggesting that even low radiation dosages might be deleterious for such individuals, increasing their risk for breast cancer or inducing severe complications. Many clinical studies have tested the hypothesis that *ATM* heterozygote breast cancer patients exhibit enhanced radiosensitivity; yet, most of those studies failed to show a clear association between *ATM* mutation status and increased radiosensitivity (Ramsay et al. 1996; Appleby et al. 1997; Ramsay et al. 1998; Weissberg et al. 1998; Shayeghi et al. 1998). Iannuzzi et al. (2002) reported that breast cancer patients harboring *ATM* missense mutations are more susceptible to develop subcutaneous late responses after radiation therapy. Using an ATM kinase assay, Scott et al. (2002) demonstrated that only 1 of 5 missense mutations detected in breast cancer patients abrogated ATM kinase activity when expressed in AT1ABR cells. The ATM protein is a large serine-threonine protein kinase that plays a key role in the cellular response induced by ionizing radiation by directly interacting and phosphorylating key components of different DNA damage response pathways such as TP53, MDM2, and BRCA1. Therefore, we cannot rule out the potential effect of these missense mutations on interindividual ionizing radiation sensitivity and cancer risk (Banin et al. 1998; Kastan and Lim 2000; Wang et al. 2000). Recently, Byrd et al. (2012) reported on a breast cancer patient with a severe reaction to radiotherapy who was a carrier of biallelic *ATM* mutations, including a missense mutation c.8672C > A (p.Gly2891Asp), and displayed residual ATM kinase activity. The expression of a missense mutated ATM protein, especially resulting from a non-conservative missense mutation, may alter the ATM protein stability and function (Gatti et al. 1999). In addition, low levels of ATM protein expression have been associated with severe adverse normal tissue reaction in breast cancer patients, suggesting that ATM protein levels may represent an independent predictive biomarker for clinical radiosensitivity (Fang et al. 2010).

The limitations of the present study are the small number of samples and low sensitivity of the methodology; although, we identified several *ATM* polymorphisms in both breast cancer patients and the control population. In an effort to better understand the etiology and to identify factors associated with the predisposition to breast cancer, several groups have recently used next-generation sequencing (NGS) to determine the pattern of mutations associated with the disease. Despite the large number of studies using NGS, no investigation has specifically focused on the *ATM* gene. Studies using exome sequencing technologies in breast cancer showed a high prevalence of mutations and variants in genes such as *BRCA1*, *BRCA2*, *TP53*, and *PIK3CA*, but few reported findings for *ATM* (Network CGA 2012; Banerji et al. 2012). A study evaluating 691 sporadic breast tumors, identified mutations affecting genes such as *PIK3CA*, *FGFR2*, *TP53*, and *ERBB2*, but no alterations in the *ATM* gene (Wilkerson et al. 2014). On the other hand, studies evaluating hereditary breast cancer have identified mutations and variants of *ATM* in a subset of cases (Snape et al. 2012; Kurian, et al. 2014). Thus, at present, data related to the *ATM* gene in sporadic breast tumors are limited; thus, it is difficult to make an adequate assessment of its role in sporadic breast cancer. Our group recently analyzed exome sequencing data from breast cancer samples, including one of the cases (M294) included here, and our preliminary (unpublished) results identified a larger number of ATM alterations compared with the data obtained from SSCP screening and direct DNA sequencing. Using SSCP analysis, we identified two missense variants in case M294 (p.L895L and p.G2023R); however, using exome sequencing, we found five missense variants (p.Y731Y, pP872S, pL895L, p.N1983S, and p.G2023R) and many INDELs and base substitutions in the introns of the ATM gene in this same case (Additional file 2). Thus, the NGS technique is a promising approach to better estimate the number of ATM variants related to breast cancer.

## Conclusions

Our results support the hypothesis that *ATM* heterozygotes carrying low penetrance *ATM* mutant alleles are at risk for breast cancer. There is a potential increased sensitivity of these heterozygotes to ionizing radiation; therefore, identifying these carriers may have an impact on the treatment offered to these individuals. We have identified several *ATM* variants in this study that may potentially act as disease-causing missense mutations. However, further functional studies to evaluate the biological effects of the potential *ATM* missense mutations observed in breast cancer patients and large case–control studies using NGS (amplicon and/or exome sequencing) are needed. These investigations should contribute to the understanding of the role played by the *ATM* gene in the etiology and pathobiology of breast cancer.

## Materials and methods

### Samples

Paired normal and tumor DNA samples were obtained from 100 sporadic breast cancer patients with no family history of A-T at Hospital do Cancer A.C. Camargo, São Paulo, Brazil (from 1993 to 1998). The age of the patients at the time of operation ranged from 25 to 78 years (median 48 years). Tumor samples were dissected to remove residual normal tissue before freezing and stored in liquid nitrogen. The largest diameter of the tumors was recorded. The number of lymph node metastases was determined by microscopic examination of an average of 24 lymph nodes per patient. Tumor metastasis to the lymph nodes was detected in 58 patients. All cases were submitted for histopathological review of tumor sections to confirm the diagnosis. All tumors were classified as invasive ductal carcinomas according to the World Health Organization Histological Typing of Breast Tumors classification, and the clinical disease stage of each patient was determined according to the 5th Edition of the UICC TNM classification of malignant tumors. In addition, DNA was extracted from one hundred control blood samples from healthy and unrelated women at Hemocentro-Hospital das Clínicas da Faculdade de Medicina da Universidade de São Paulo. The Institutional Ethics Committee approved this study and all subjects studied (patients and matched controls) signed an informed consent for participation*.*

### DNA extraction

Normal and tumoral tissue was ground to a powder using a frozen tissue pulverizer (Termovac). The powder was resuspended in 1 mL of lysis buffer (10 mM Tris–HCl, pH 7.6, 1 mM EDTA, and 0.6% SDS) with 100 μg/mL proteinase K, and incubated at 37°C overnight. High molecular weight DNA was extracted with phenol-chloroform and precipitated with ethanol. Blood samples were collected in EDTA (5 mL) and DNA was extracted as described by Lahiri & Nurnberger (1991) and adapted by Salazar et al. (1998).

### Loss of heterozygosity (LOH) analysis

The LOH analysis for the chromosomal region 11q23 was performed using three polymorphic microsatellite markers (D11S897, D11S1818, and D11S2000). Primer sequences for these markers were obtained from the Genome Data Base (http://www.ncbi.nlm.nih.gov/probe/). Allelic loss assays were performed as previously described (Miracca et al. 1999). Allelic loss, determined by densitometric scan (UltroScan XL; Pharmacia), was considered to be complete or partial if the intensity of one allele was reduced by at least 40% in the tumor compared with normal DNA samples from the same patient. LOH was scored for informative (heterozygous) patients only.

### Mutation analysis

Mutation analyses were performed by Single Strand Conformation Polymorphism (PCR-SSCP) and direct DNA sequencing analysis, as previously described (Miracca et al. 1999). Briefly, sixty-four sets of primers were used to amplify exons 2 to 63 of the *ATM* gene, as previously described by Vorechovsky et al. (1996). Radiolabeled PCR reactions were performed in 25 μL volumes using 50–100 ng of genomic DNA template, containing 0.1 mCi of [α^32^P-dCTP] (Amersham, specific activity, 3000 Ci mmol^−1^). Amplification products were diluted ten-fold in a buffer containing 95% formamide, 20 mM EDTA, 0.05% bromophenol blue, and 0.05% xylene cyanol, heated at 83°C for 5 min, and applied (3 μL per lane) on two 6% polyacrylamide non-denaturing gels, one containing 5% glycerol and the other 10% glycerol. Electrophoresis was performed at 6 W for 14–16 h at room temperature with two cooling fans. Band shift mobility was detected by autoradiography of dried gels using Kodak X-Omat XAR film with an intensifying screen for 12–48 h at −70°C. DNA samples with evidence of sequence variants in the SSCP analysis were characterized by direct sequencing using the Big Dye Terminator kit (Life Technologies™) according to the manufacturer’s specifications. Sequencing reaction products were purified by ethanol/EDTA precipitation and resuspended in Hi-Di™ formamide (Life Technologies™). Samples were separated by electrophoresis on the ABI Prism 3730 DNA Analyzer (Life Technologies™) and the results were evaluated in Mutation Surveyor^R^ DNA Variant Analysis Software version 3.30 (Softgenetics LLC). The variants identified were evaluated using the following public ATM databases to determine if changes were novel or already known: http://chromium.liacs.nl/LOVD2/home.php?select_db=ATM; NCBI dbSNP - http://www.ncbi.nlm.nih.gov/projects/SNP/snp_ref.cgi?geneId=472; Ensembl - http://www.ensembl.org/Homo_sapiens/Info/Index; and EVS - http://evs.gs.washington.edu/EVS/. In addition, the algorithms PolyPhen-2 (http://genetics.bwh.harvard.edu/pph2/) and SIFT (http://provean.jcvi.org/genome_submit_2.php) were used to predict the possible impact of amino acid changes on the function of the ATM protein.

## Additional files

Additional file 1:
**Representative example of the sequencing analysis of a breast cancer patient harboring both variants p.F858L and p.P1054R.**
Additional file 2:
**Summary of the ATM variants observed by exome sequencing analysis in Tumor and Normal tissue of one breast cancer patient (M294) Chromosomal coordinates of the ATM variants observed by exome sequencing analysis of Tumor and Normal tissues from one breast cancer patient (M294).** The results were analyzed using the Golden Helix GenomeBrowser (www.goldenhelix.com/GenomeBrowse/).
