# Supplementary material for: ATM gene mutations in sporadic breast cancer patients from Brazil
Source: Springerplus. 2015 Jan 15;4:23. doi: 10.1186/s40064-015-0787-z (PMC4298590; doi:10.1186/s40064-015-0787-z)
Supplement: Additional file 1: — Representative example of the sequencing analysis of a breast cancer patient harboring both variants p.F858L and p.P1054R. [file 40064_2015_787_MOESM1_ESM.pdf]

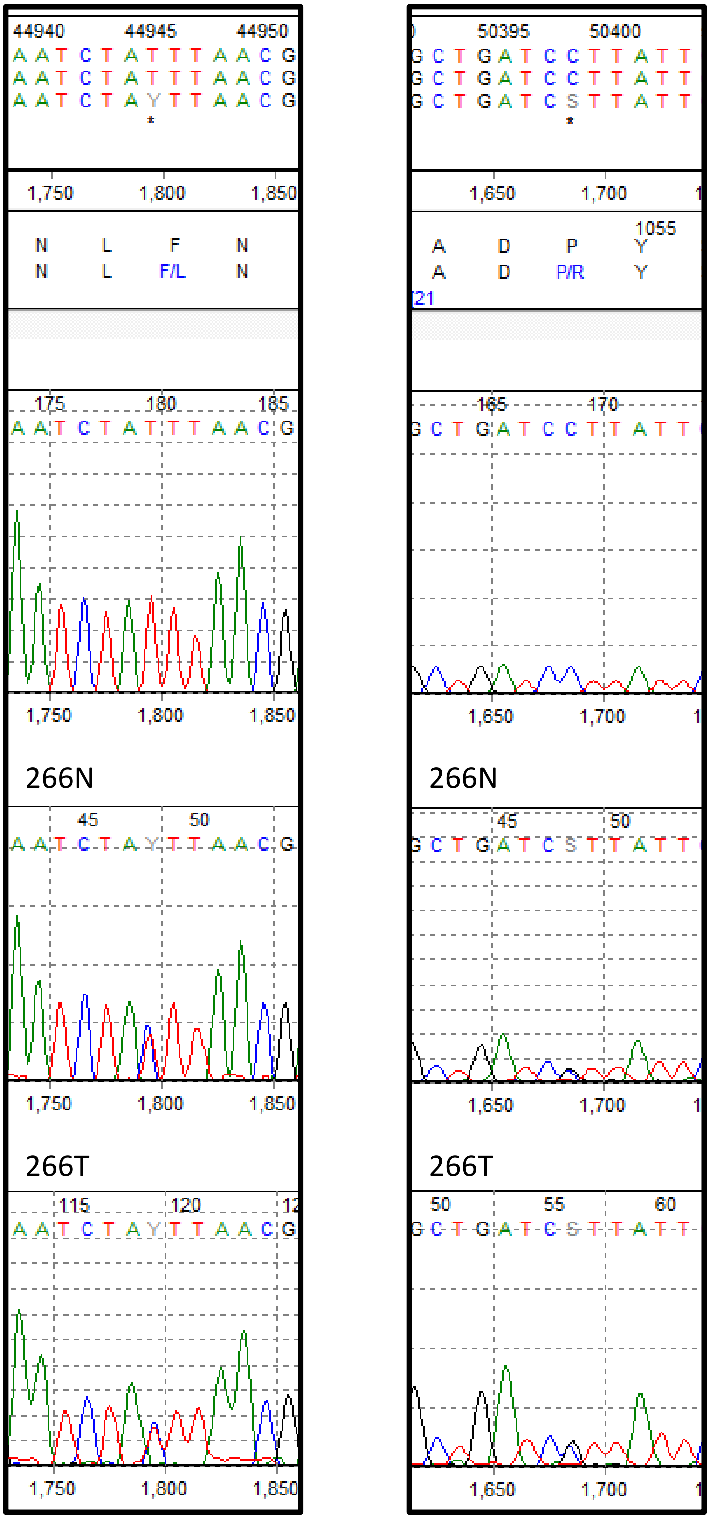


Exon 19
c.2572T>C
p.F858L

Exon 24
c.3161C>G
p.P1054R

Supplementary Material 1. Representative example of the sequencing analysis of a breast cancer patient harboring both variants p.F858L and p.P1054R.
