# Supplementary material for: ATM gene mutations in sporadic breast cancer patients from Brazil
Source: Springerplus. 2015 Jan 15;4:23. doi: 10.1186/s40064-015-0787-z (PMC4298590; doi:10.1186/s40064-015-0787-z)
Supplement: Additional file 2: — Summary of the ATM variants observed by exome sequencing analysis in Tumor and Normal tissue of one breast cancer patient (M294) Chromosomal coordinates of the ATM variants observed by exome sequencing analysis of Tumor and Normal tissues from one breast cancer patient (M294). The results were analyzed using the Golden Helix GenomeBrowser (www.goldenhelix.com/GenomeBrowse/). [file 40064_2015_787_MOESM2_ESM.pdf]

Summary of the ATM variants observed by exome sequencing analysis in Tumor and Normal tissue of one breast cancer patient (M294)

Position Exon/Intron	Nucleotide Change	Residue	N. altered reads/total N. of reads		Genomic Coordinate
			294N	294T	
Intron 7-8	delT		21/98	14/92	108114662
Intron 8-9	delT		13/15	21/21	108115361
Intron 13-14	G>A		26/54	19/32	108123290
16	TAC>TAT	p.Y731Y	60/104	61/116	108127010
19	CCT>TCT	p.P872S	16/38	25/39	108138045
Intron 19-20	delT		11/23	12/30	108139043
20	CTA>CTG	p.L895L	9/24	9/29	108139183
Intron 22-23	C>T		81/154	55/101	108143182
Intron 25-26	insA		40/80	39/76	108151707
intron 36-37	T>C		20/ 45	37/ 53	108172225
42	AAT>AGT	p.N1983S	198/198	125/125	108183167
43	GGA>AGA	p.G2023R	54/98	31/57	108186610
Intron 43-44	T>C		37/89	20/ 46	108186653
Intron 47-48	G>T		4/8	8/17	108192159
Intron 48-49	G>C		4/ 8	8 /17	108196509
Intron 55-56	G>A		69/69	51/51	108203769
Intron 56-57	INS T		47/106	37/ 85	108204717
Intron 59-60	A>G		13/21	8/ 18	108214176
Intron 61-62	T>C		30/63	17/ 29	108218196
Intron 62-63	C>T		42/79	38/ 61	108225483
Intron 63-64	A>G		15/30	21/ 31	108225661

Chromosomal coordinates of the ATM variants observed by exome sequencing analysis of Tumor and Normal tissues from one breast cancer patient (M294). The results were analyzed using the Golden Helix GenomeBrowser (www.goldenhelix.com/GenomeBrowse/)

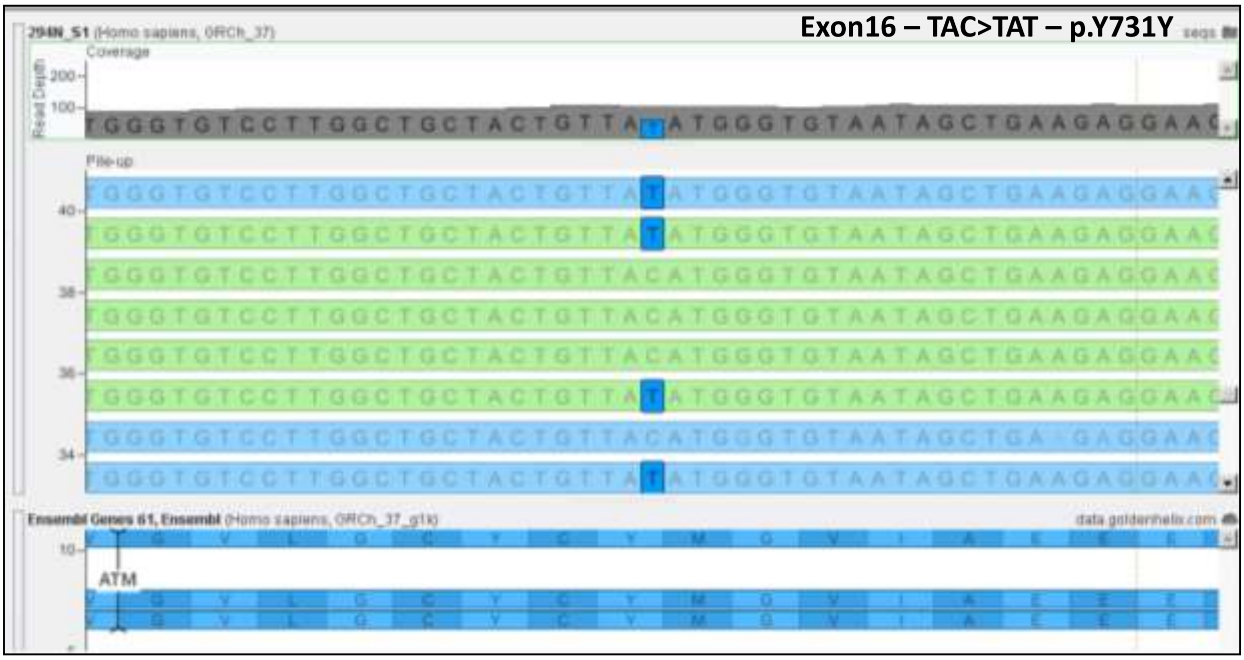

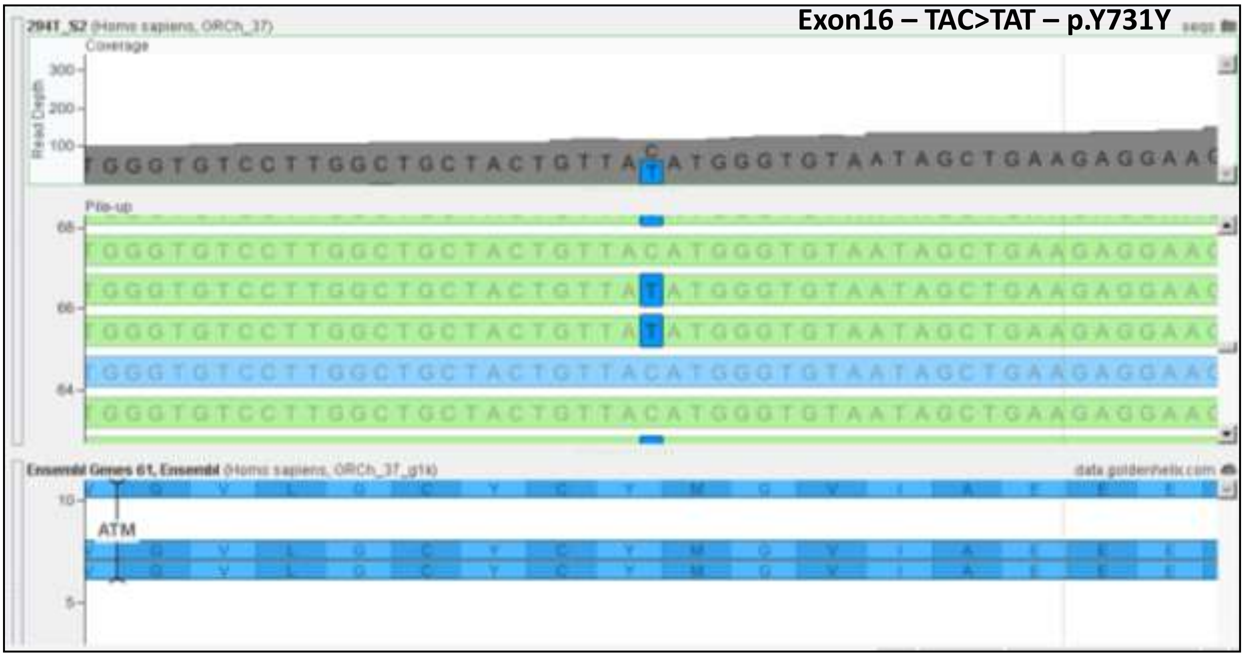

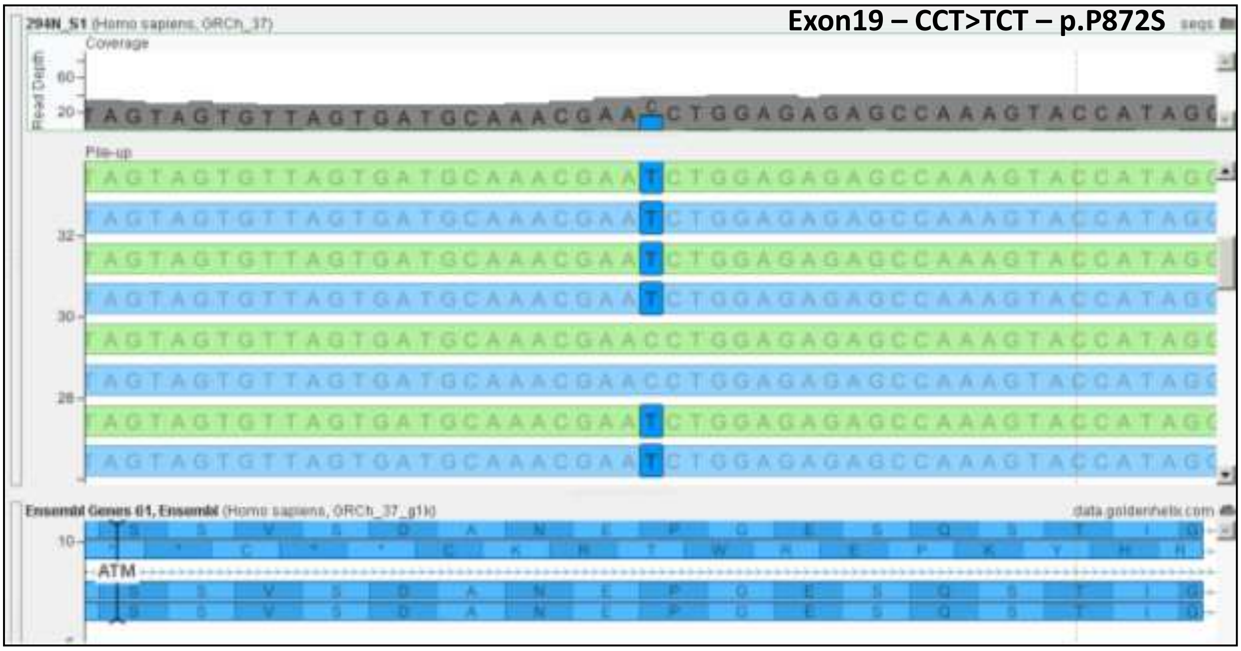
Exon19 – CCT>TCT – p.P872S
294N_S1 (Homo sapiens, GRCh_37)
Coverage
Read Depth
Pile-up
Ensembl Genes 61, Ensembl (Homo sapiens, GRCh_37_g1k)
data.goldenhelix.com
ATM

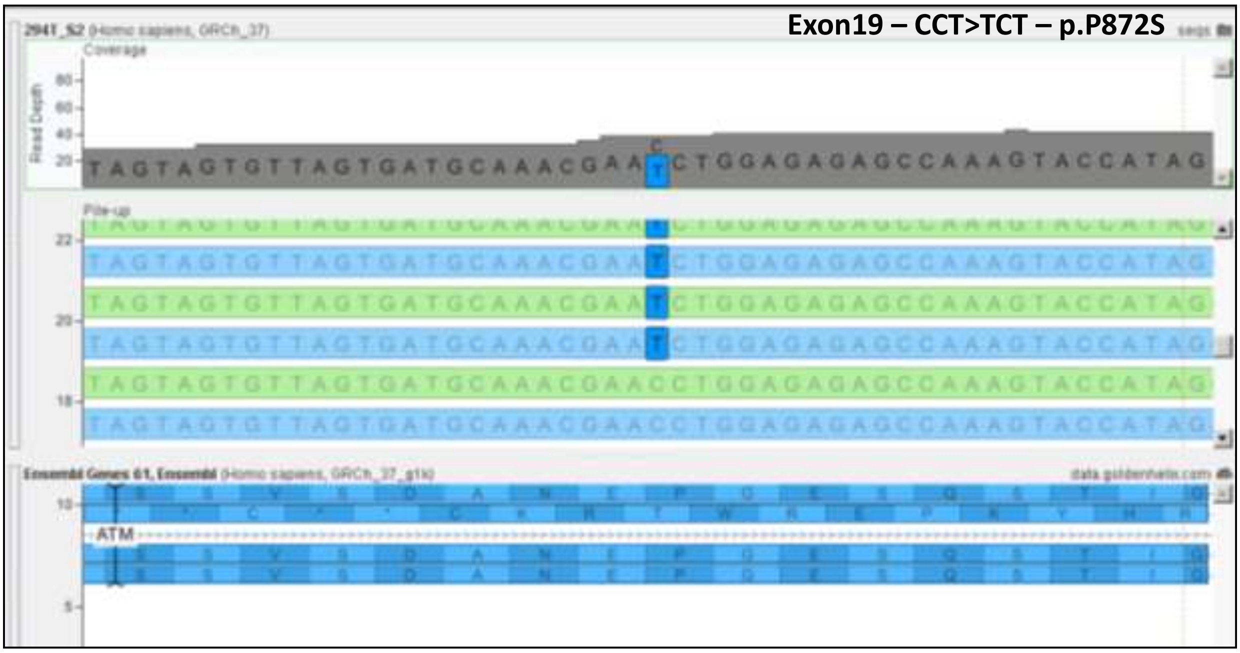
Exon19 – CCT>TCT – p.P872S
294T_S2 (Homo sapiens, GRCh_37)
Coverage
Read Depth
Pile-up
Ensembl Genes 61, Ensembl (Homo sapiens, GRCh_37_g1k)
data.goldenhelix.com
ATM

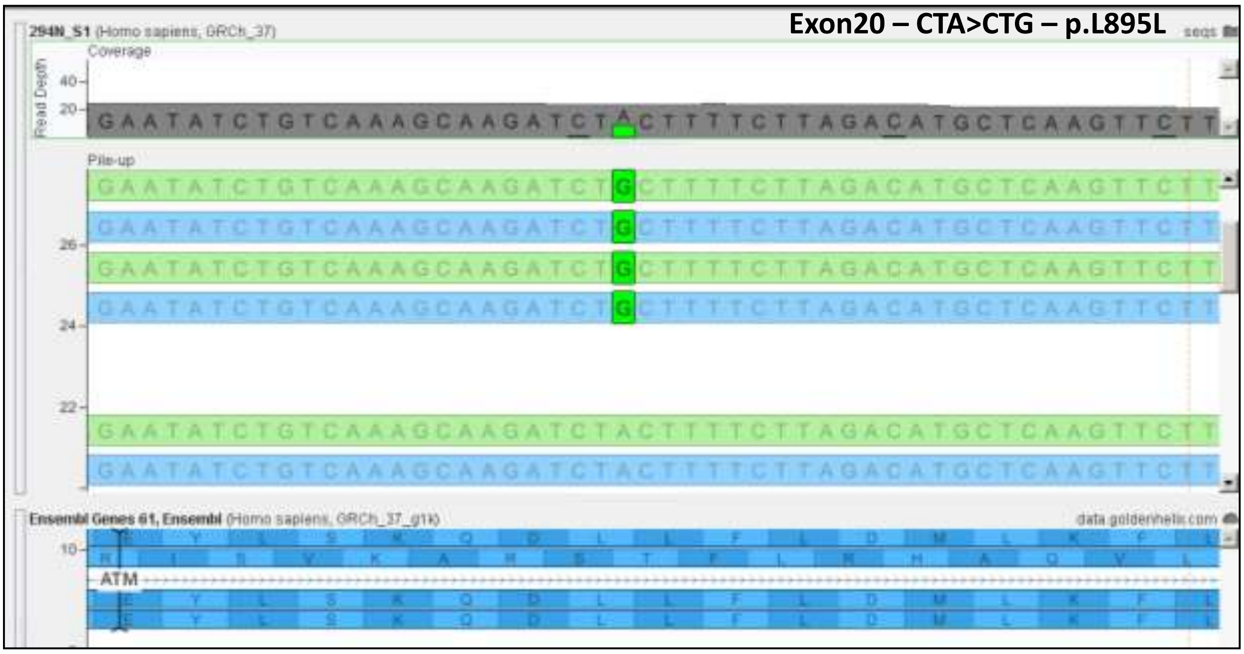
Exon20 – CTA>CTG – p.L895L
294N_S1 (Homo sapiens, GRCh_37)
Coverage
Read Depth
GAATATCTGTCAAAGCAAGATCTACTTTTCTTAGACATGCTCAAGTTCTT
Pile-up
Ensembl Genes 61, Ensembl (Homo sapiens, GRCh_37_g1k)
ATM
data.goldenhelix.com

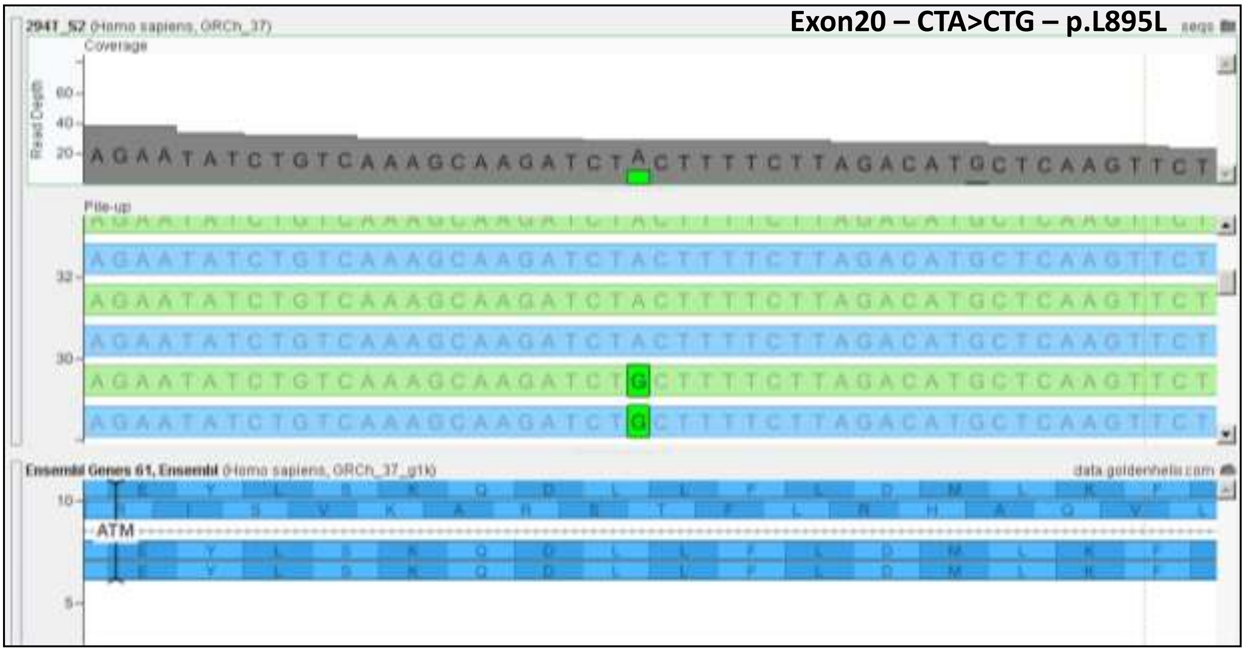
Exon20 – CTA>CTG – p.L895L
294T_S2 (Homo sapiens, GRCh_37)
Coverage
Read Depth
AGAATATCTGTCAAAGCAAGATCTACTTTTCTTAGACATGCTCAAGTTCT
Pile-up
Ensembl Genes 61, Ensembl (Homo sapiens, GRCh_37_g1k)
ATM
data.goldenhelix.com

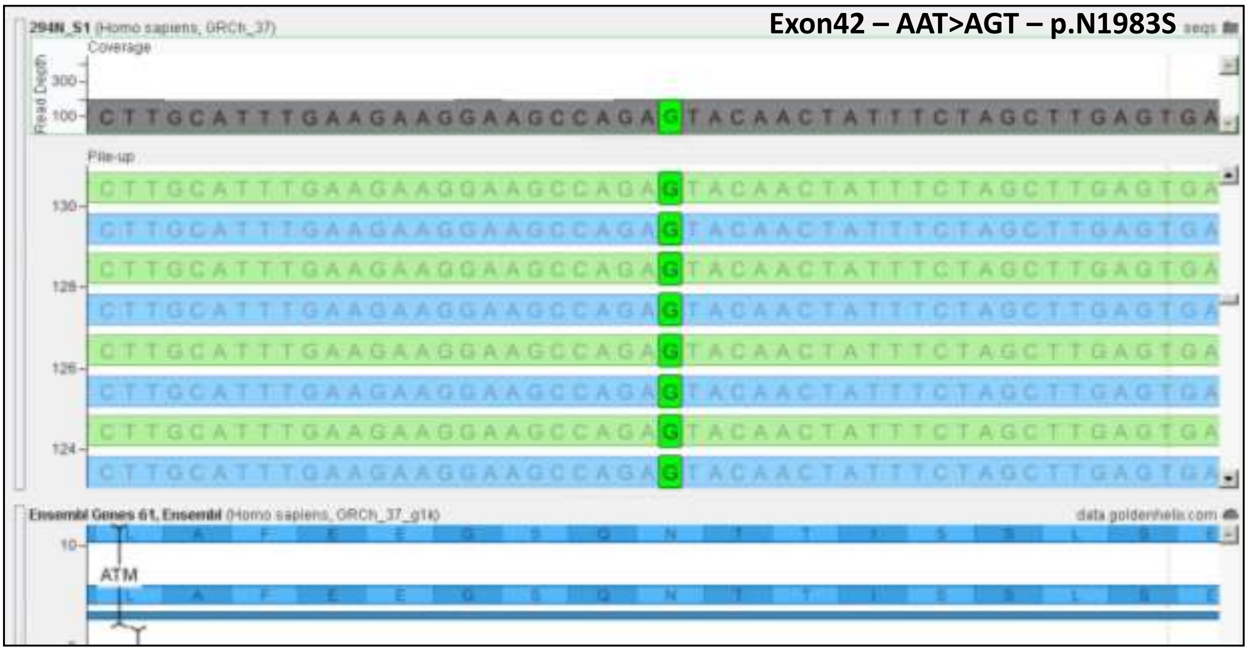
294N_S1 (Homo sapiens, GRCh_37)
Exon42 – AAT>AGT – p.N1983S
Coverage
Read Depth
Pile-up
CTTGCATTTGAAGAAGGAAGCCAGAGTACAACTATTTCTAGCTTGAGTGA
Ensembl Genes 61, Ensembl (Homo sapiens, GRCh_37_g1k)
data.goldenhelix.com
ATM

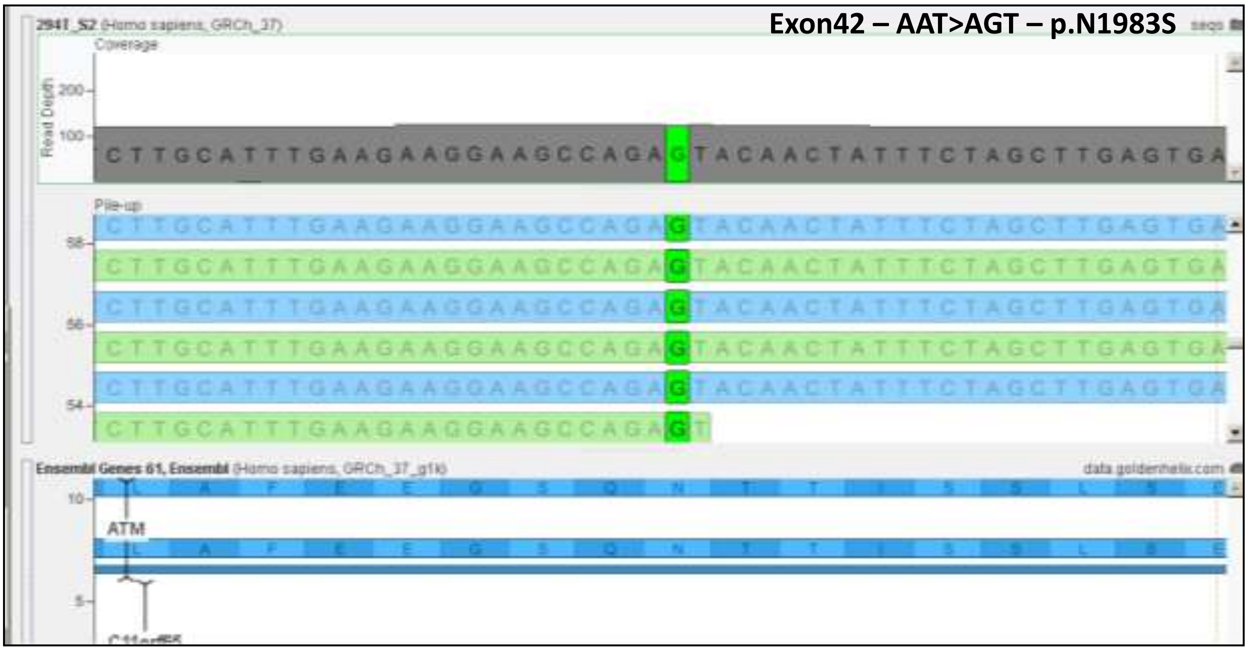
294T_S2 (Homo sapiens, GRCh_37)
Exon42 – AAT>AGT – p.N1983S
Coverage
Read Depth
Pile-up
CTTGCATTTGAAGAAGGAAGCCAGAGTACAACTATTTCTAGCTTGAGTGA
Ensembl Genes 61, Ensembl (Homo sapiens, GRCh_37_g1k)
data.goldenhelix.com
ATM

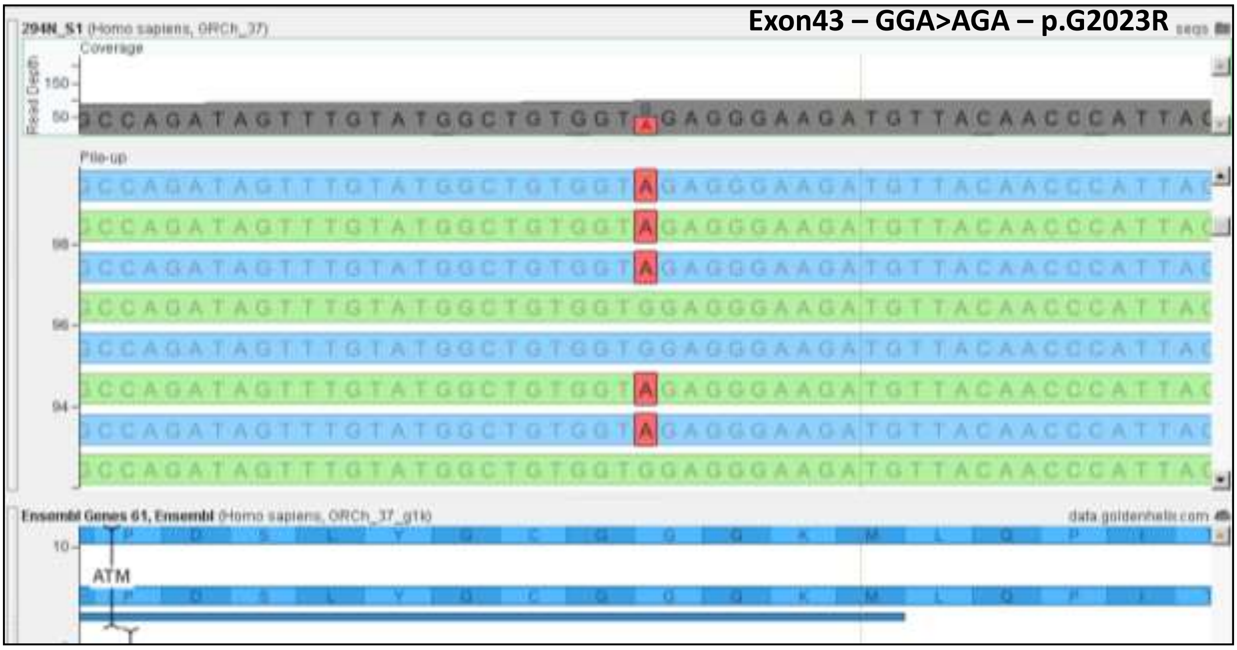
Exon43 – GGA>AGA – p.G2023R
294N_S1 (Homo sapiens, GRCh_37)
Coverage
Read Depth
Pile-up
Ensembl Genes 61, Ensembl (Homo sapiens, GRCh_37_g1k)
data.goldenhelix.com
ATM

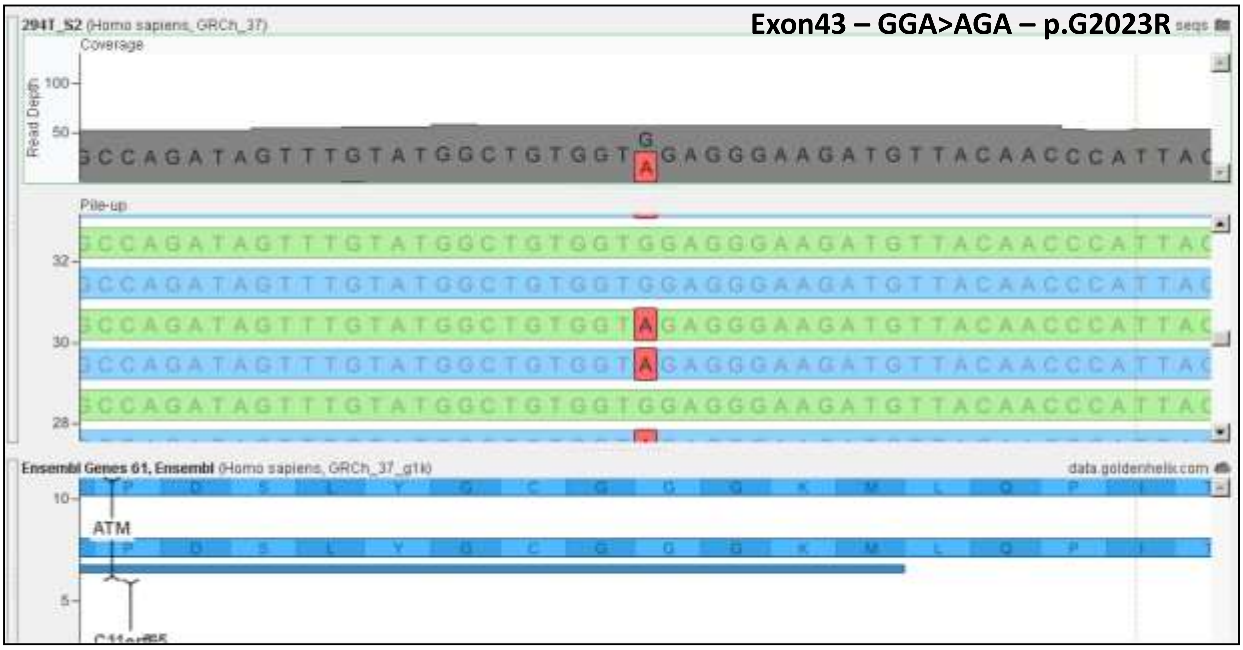
Exon43 – GGA>AGA – p.G2023R
294T_S2 (Homo sapiens, GRCh_37)
Coverage
Read Depth
Pile-up
Ensembl Genes 61, Ensembl (Homo sapiens, GRCh_37_g1k)
data.goldenhelix.com
ATM
